# Nail Cancer: Review of the Two Main Types of an Underestimated Disease

**DOI:** 10.7759/cureus.23856

**Published:** 2022-04-05

**Authors:** Camilo Levi Acuña Pinzon, Jefferson Fabian Nieves Condoy, Daniel A Rivera Marquez, Alan Ramón Javier Collazo Moreno, Roland Kevin Cethorth Fonseca, Luis Abraham Zúñiga Vázquez

**Affiliations:** 1 Surgery, Hospital Regional de Alta Especialidad del Bajío, León, MEX; 2 General Surgery, Hospital Regional de Alta Especialidad del Bajío, León, MEX; 3 Surgical Oncology, Hospital Regional De Alta Especialidad Del Bajío, León, MEX

**Keywords:** malignant tumors, malignant tumors of nail, nail apparatus, nail disease, melanoma, squamous cell carcinoma, nail cancer

## Abstract

Neoplastic lesions (benign or malignant) in the nail region are rare when compared to lesions in the rest of the skin. Despite advances in diagnostic modalities, their diagnosis is frequently delayed or overlooked for days, months, or even years when they are misrecognized or when their approach is not appropriate. Undoubtedly, malignant tumors are the most important lesions since an inopportune diagnosis or treatment can drastically change the patient's prognosis.

A review of all the scientific evidence on the two main malignant neoplasms of the nail apparatus (melanoma and squamous cell carcinoma) was carried out using the PubMed search engine from 2003 to 2022, in order to expose the appropriate diagnostic approach and treatment of these nail lesions to avoid delays that obscure the prognosis of patients. This review does not include reconstruction modalities after lesion resection, but the emphasis is placed on the great functional impact they produce. Surgical treatment in the early stages is the most important when talking about prognosis and emphasizing it; systemic oncological management of advanced stages is not so deep.

## Introduction and background

The nail apparatus is a fundamental functional and aesthetic part of the digital area. Despite being rare, all the cells that make up its tissues can give rise to neoplastic lesions [1]. Nail neoplasms include benign and malignant tumors that have different signs and symptoms causing deformities and affecting nail growth. In general, malignant tumors deform the adjacent tissue while benign tumors preserve tissue architecture [2], however, it should not be considered a rule.

Malignant tumors are the most important lesions of the nail apparatus for their impact on the prognosis [1]. Among the malignant neoplasms of the nail apparatus, we find squamous cell carcinoma and melanoma; melanoma represents 0.18% to 7% of cutaneous melanomas [2] and squamous cell carcinoma has a prevalence of 0.0012% to 0.028% (varies depending on tumor subtype and its causes) [3]. Cases of the coexistence of squamous cell carcinoma and melanoma have been described [4].

A biopsy is the only confirmatory diagnostic means. In general, three conditions must be met to achieve an accurate diagnosis [5]. Collect the sample properly and provide the pathologist with information on the medical history, proper handling of the sample in the pathology laboratory (which includes orientation of the sample before embedding in paraffin, adequate sections, and softening of the sample if the nail plate is present), and the pathologist must know the normal histology of the nail. Failure to properly orient the sample, a common error, prevents proper evaluation of the edges and subsequently complicates the choice of surgical treatment. Technical recommendations for taking biopsies are mentioned later.

## Review

Methods

The PubMed search engine was used to perform a search using the keywords “nail”, “nail cancer”, “nail melanoma”, “squamous cell carcinoma”, “melanoma”, “nail squamous cell carcinoma.” to find articles published from 2003 to 2022 on the epidemiology, diagnosis, and treatment of nail cancer. 2003 was chosen as the initial period for the search in order to synthesize current information from the last two decades.

Reviews, case series, case reports, and international guidelines were obtained. All articles that mentioned important aspects of the pathology according to the authors' own criteria were included. Once all the articles to be reviewed were obtained, a round table was held in order to discuss the most relevant information to include in our narrative review.

Review

We consider that for an adequate understanding of malignant neoplasms of the nail, a deep knowledge of the anatomy of this region is necessary. Since a deep anatomical study is not the purpose of our review, we proceed with a brief description of this area. Solis et al. give an excellent description of this anatomy, and we refer our readers to this publication for a more detailed description [6].

**Figure 1:**
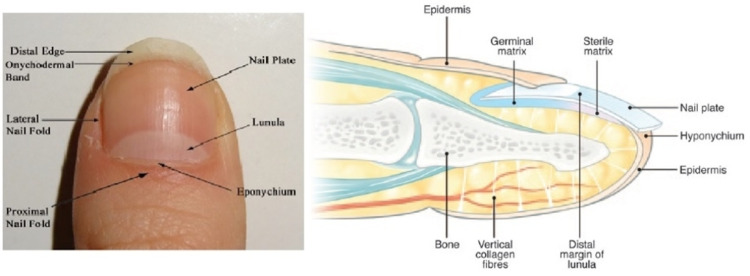
Nail anatomy Left: Surface anatomy of the nail apparatus. Right: Sagittal view of the nail apparatus. (Image taken from Solish D, Weinberg T, Murray C: Surface Anatomy of the Nail for the Dermatologist. J Cutan Med Surg. 2016, 20:467-9 with pre-approved permission of the Journal of Cutaneous Medicine and Surgery.)

The nail apparatus is composed of epithelial tissues (nail matrix, nail bed, and nail plate) and mesenchymal tissues (underlying dermal tissue and onychodermis, the latter constituting specialized mesenchyme containing onychofibroblasts located under the nail matrix) (Figure 1) [6].

The most prominent central rectangular structure is the nail plate; it firmly adheres to the germ and sterile matrices that make up the nail bed. The proximal quarter of the nail plate is covered by the proximal nail fold, which has two surfaces; the cuticle or eponychium is at the junction of these two surfaces. The lunula represents the distal part of the germinal nail matrix and is known as the "white crescent." The hyponychium is an epithelium located at the junction of the free edge of the nail plate and the skin; together with the eponychium, the hyponychium forms a barrier against pathogens. The transition between the nail bed and the hyponychium is called the onychodermal band. The nail matrix is divided into germinal and sterile, the latter is distal to the lunula. The germinal matrix is located proximal to the distal edge of the lunula and it has two portions, a proximal portion that lies below the proximal nail fold and cuticle and a distal portion that is visible on the surface as the lunula [6].

Melanoma

Nail melanoma is a rare variant, with an incidence of 0.31% to 1.5% in the Caucasian population and with a relative frequency of up to 20% in Africans and Asians [7]. It is responsible for the higher mortality from nail cancer [8]. Although pediatric cases have been described, the peak incidence is in the sixth decade of life [7]. It occurs most frequently in the first finger of both feet and hands and more infrequently in the fifth finger in both regions [9-10]. Although trauma, exposure to ultraviolet light, or family history have been proposed as risk factors, its etiology remains unknown [7,11]. Mutations have been identified in the BRAF, NRAS, KIT, MAP2K2, and NF1 genes [11].

For years, the ABCD (asymmetry, border, color, and diameter) rule has been applied as part of the diagnosis of melanomas [11]; unfortunately, the anatomical complexity of the nail apparatus does not allow its proper application. Levit et al. proposed a specific acronym ABCDEF for melanoma of the nail apparatus, in which it relates age (Range 20-90 years with a peak in the 5th-7th decades), the presence or absence of pigmented bands (Breadth >3 mm and irregular border), changes in size or lack of improvement with treatment, the compromised finger (thumb > hallux index, single digit > multiple digits), the extension (Hutchinson´s sign), and family history (previous melanoma or dysplastic nevus syndrome) [12].

Longitudinal melanonychia, defined as a brown or black stripe on the nail bed, is caused by hyperplasia or the activation of melanocytes. Although it can be caused by subungual hematomas, bacterial infections, Onychomycosis nigricans, human immunodeficiency virus (HIV), metabolic deficits, endocrine disorders, medications, radiation, and benign tumors; it is also the first sign of melanoma and it occurs in approximately 70% of cases. Width or diameter of the lesion greater than 3 to 6 mm correlates with a greater risk of malignancy, and if the band is wider proximal than distal, it usually indicates a rapidly growing melanoma. If an infection is suspected, and there is no response after several months of treatment, a biopsy should be obtained to rule out neoplasia. The typical mode of spread is from the nail matrix directly to the adjacent nail plate and through the nail bed to the hyponychium and nail folds, explaining the presence of Hutchinson's sign (extension of brown or black pigmentation in the adjacent periungual skin) in the nail folds and even on the fingertip [13].

Dermoscopy is a non-invasive procedure that uses magnification, illumination, and gel as a means of evaluating the areas of pigmentation and the characteristics of the lesion, allowing a more microscopic view of the structures not visible to the naked eye. Irregular brown or black bands, non-parallel lines, poorly defined borders, width greater than 3 mm, dystrophy, ulceration, and Hutchinson's sign are highly suggestive of melanoma and allow a distinction to be made with benign processes [13]. Melanomas often extend beyond the visible margin of the lesion, which is particularly important because there is very little subcutaneous tissue between the skin and the bone [13].

Although the biopsy may miss a malignant lesion or be inconclusive, it is considered essential for diagnosis. Several biopsy techniques have been described, including scraping or puncture methods. Littleton et al. recommend full-thickness longitudinal excisional biopsies of pigmented areas, including nail folds or fingertip skin [13].

The histopathological progression of melanoma includes benign nevi, radially growing melanoma in situ, invasive vertically growing melanoma, and finally metastatic melanoma spreading through the lymphatic system [13]. Kerl et al. propose to differentiate nevi from malignant melanoma by the presence of subtle findings such as numerous melanocytes and melanin granules, diffusely distributed melanin, and atypical melanocytes (pleomorphism, cellular atypia, and mitosis) [14]. Histopathologically, the subtypes of melanoma of the nail apparatus are acral lentiginous, nodular (20-39%), and superficial dissemination (8-27%) [10]. Immunohistochemistry can be very useful; MART 1 or HMB 45, SOX 10, and protein S100 positive with negative cytokeratin are considered to confirm the diagnosis of melanoma [13] but are considered imprecise as markers of subungual melanoma. Upregulation of preferentially expressed antigen in melanoma (PRAME) has been reported to be a common event in melanomas, and relatively recently, immunohistochemistry for PRAME has been shown to be diagnostically helpful in evaluating various melanocytic lesions [15].

The initial treatment is resection of the lesion, for which we have several options: Mohs surgery, wide local excision, and amputation. Given the close relationship between the nail matrix and bone, Breslow's depth criteria are of little use. Mohs surgery is recommended for melanomas in situ, which are defined as a tumor that does not involve the entire depth of the nail matrix. Disease-free survival at five and 10 years of 92% and 83%, respectively, has been described with this technique; however, there are still no major series and some results are uneven. Historically, amputation has been the treatment of choice but there is still controversy about the level of amputation and the impact that the loss of a finger (for example, the thumb) entails on the quality of life of the patient. Wide local resection has been proposed, resecting the entire lesion and allowing posterior coverage of the defect [13].

According to Ogata et al., there is no difference in disease-free survival and overall survival between the different types of procedures, and it is recommended to consider tissue-sparing procedures depending on the tumor volume to be resected [9]; however, since the recommendations for cutaneous melanoma cannot be applied to the nail plate due to its special anatomy, it is considered that there is insufficient evidence to recommend a resection margin or conservative procedures over amputation [13].

In general, nail melanoma has a worse prognosis than other melanomas. Higher rates of positive sentinel node have been reported in tumors of the nail apparatus, reflecting its locally advanced nature at the time of diagnosis; however, late diagnosis, insufficient biopsy material, or diagnostic error, especially in the early stages, have been discussed as causes of its gloomy prognosis [7].

Sentinel node biopsy (SLNB) provides additional information for management and treatment [7]. In general, SLNB is not recommended for T1a patients since the risk of being positive is only 5%. If the patient is T1b, they have a 5% to 10% risk and SLNB should be considered but if the risk is greater than 10% (patient T2a - T4b), SLNB should be offered to all patients [16]. The study of the patient should be completed with a tomography or magnetic resonance imaging of the skull, thorax, abdomen, and pelvis, and positron emission tomography (PET)-computed tomography (CT) if it is available [7].

The National Comprehensive Cancer Network (NCCN) guidelines for cutaneous melanoma mention several points regarding the treatment of unresectable or metastatic disease. Nivolumab + ipilimumab has traditionally been recommended as first or second-line treatment but has been associated with high rates of toxicity. Low doses of ipilimumab have been proposed as well as associating pembrolizumab with low doses of ipilimumab, however, equivalence studies on these regimens are still required. For patients with the BRAF V600 mutation, therapy with BRAF/MEK inhibitors has responses of 67%-70% with a median duration of 12 to 18 months. Triple therapy regimens combining BRAF, MEK, and PD 1 (vemurafenib/cobimetinib/atezolizumab or dabrafenib/trametinib/pembrolizumab) have recently been proposed as they improve the duration of response [16].

Squamous cell carcinoma

Squamous cell carcinoma of the nail apparatus has a prevalence of 0.0012% to 0.028%; it can occur at any age but has a peak incidence between 50 and 69 years. The male-female ratio is 2 to 1. It generally involves only one finger; the thumb being the most frequently affected (44%) [3].

Depending on the histological characteristics, we can classify squamous cell carcinoma as low/moderate risk (keratoacanthomas, warty carcinoma, clear cell carcinoma) and high risk (acantholytic carcinoma, spindle cell carcinoma, and adenosquamous carcinoma). Some variants require the use of immunohistochemistry for their characterization, among which we find carcinoma with sarcomatoid differentiation, lymphoepithelioma-like carcinoma, and giant cell carcinoma [17].

Among the main risk factors, we can find trauma, chronic exposure to sunlight and arsenic, radiation, burns, genodermatosis, smoking, immunosuppression, and human papillomavirus infection [3]. Knowing the risk factors is very important because the disease usually develops from a precursor lesion such as actinic keratosis, Bowen's disease, burn scars, or chronic radiation dermatitis [18-19].

Human papillomavirus (HPV) infection is a factor especially involved in the pathogenesis; it is being considered responsible in 60% to 80% of cases, especially serotype 16 (responsible in approximately 57% of cases) [19]. Other serotypes reported are 2, 6, 11, 18, 26, 31, 34, 35, 56, 58, 67, and 73 [2]. Digital self-inoculation of HPV through digital manipulation of the genitalia can play a very important role in the transmission of high-risk serotypes [19].

The typical presentation is a slow-growing, indolent lesion, or with very mild symptoms. The subungual region is the most frequent, however, there is a great variety of clinical appearances among which we find onycholysis, painless erosion without nodule, erosion with nodule, longitudinal erythronychia, synchronous carcinoma, and hyperkeratosis. It tends to be invasive with bone involvement in 16% to 66% of cases [3].

A biopsy is the only method to confirm the diagnosis, and it should be performed on any nail lesion that does not respond to medical management. Although several techniques to perform nail biopsies have been described, it is suggested prior exposure of the nail bed be performed to avoid taking inappropriate samples [20].

Onicoscopy, a non-invasive method of nail visualization, is another useful diagnostic tool. Onycholysis, irregular vascularity, area of hemorrhage, and rough warty surface are the main findings in this diagnostic modality, however, these findings must be interpreted with care because none are pathognomonic of the disease and make it necessary to carry out a differential diagnosis. It has been shown to decrease the number of unnecessary resections [3].

Confocal fluorescence microscopy is a recent modality in which the finding of cytological and structural atypia, nuclear pleomorphism, and irregular and clustered nucleus suggest the diagnosis. It is used intraoperatively to confirm the diagnosis and evaluate the surgical margins, and it can become an alternative method to classical Mohs surgery [3], but more studies are required to assess its effectiveness and cost-effectiveness.

Among the diagnostic images, we can find radiography, ultrasound, tomography, and magnetic resonance. Bone compromise demonstrated by plain radiography indicates the need for amputation [19], however, it is only possible to observe this compromise in less than 20% of cases [3]. Despite the low performance of radiography, we believe that it is a rapid and widely available method for assessing bone extension and should be performed in all patients unless they have already been studied with another imaging method.

Ultrasound will show a heterogeneous hypoechoic focal mass with irregular margins and signs of low pulsatile resistance with the use of Doppler [3]. Tomography usually shows a soft tissue mass with an osteolytic defect of the phalanx without a periosteal reaction. Magnetic resonance imaging detects lesions that are not clinically evident but painful and is superior in predicting the location and extent of the tumor [3,20], however, no studies support its use routinely due to the difficulty of differentiating squamous cell carcinoma from other conditions [20]. In case of lymph node disease or suspicion of the same, chest tomography and ultrasound of the axillary and supratrochlear lymph nodes should be performed routinely [3].

The most used treatment options are resection and amputation. The main criterion for choosing the surgical procedure is the radiographic findings. In case of bone invasion, amputation of the distal phalanx or disarticulation of the affected finger should be performed. Despite the fact that disarticulation has a lower recurrence rate, the functional loss must be weighed and there is no strong recommendation about one or the other procedure [3]. In a case report on a patient with bone involvement due to squamous cell carcinoma, wide resection and periosteal resection without amputation were made with good evolution and without data of recurrence in the postoperative follow-up; more evidence is still required to perform a recommendation in this regard [21]. A resection margin between 4 and 5 mm has been recommended in some studies [3,6], but there is no good quality evidence to give a specific recommendation.

Mohs micrograph surgery allows resection with a minimal residual scar, reducing the number of amputations, saving tissue, and preserving quality of life, it also allows periosteal evaluation and compression difference due to neoplasm inflammation [2-3]. Although there are no comparative studies between Mohs surgery and other techniques, recurrence rates with Mohs surgery are considered to be significantly lower [22].

Other treatment options include radiation therapy and topical chemotherapy. Radiation therapy is useful in cases of multi-finger involvement or in cases where the surgery is technically difficult or a great functional involvement of the limb in the postsurgical. The main risk of radiotherapy is the development of cancer in the irradiated healthy tissue [3,20].

5-fluorouracil or imiquimod cream, cidofovir, or podophyllin are therapies that have been shown to have a high risk of relapse and are associated with loss of histological control of surgical margins, for which reason they are not currently recommended [3,20]. Systemic chemotherapy is only indicated in metastatic disease [3]. The National Comprehensive Cancer Network (NCCN) guidelines describe a wide variety of cytotoxic therapies that have been evaluated for regional or metastatic disease. The most commonly used are cisplatin, carboplatin, and 5-fluorouracil in monotherapy or in combination regimens. A carboplatin + paclitaxel regimen has been studied in combination with radiotherapy for squamous cell carcinoma of the head and neck with responses of 82%, disease-free survival, and overall survival at 30 years of 78.8% and 76.5%. Immunotherapy with pembrolizumab and cemiplimab is recommended for locally advanced, recurrent, or metastatic disease if radiotherapy or surgery is not possible. Radiotherapy can be used as initial treatment in patients without lymph node involvement who prefer this method or whose surgical sequelae are too great, however, recurrence rates have been reported from 2.8% to 30% [23].

## Conclusions

Neoplastic lesions of the nail apparatus are infrequent diseases but they can generate a great functional impact. Within these neoplasms, a malignant disease usually has a poor prognosis due to delayed diagnosis in primary care units.

The two main malignant pathologies in this region are squamous cell carcinoma and melanoma, diseases on which this narrative review was carried out. The treatment of local or locoregional disease is wide resection or amputation, with or without lymph node dissection, respectively. There is still no conclusive literature to give recommendations on resection margins or ideal reconstructive procedures. Systemic treatment can be proposed mainly in metastatic disease, with new treatment approaches recently available.
